# Proposal and Evaluation of Visual Haptics for Manipulation of Remote Machine System

**DOI:** 10.3389/frobt.2020.529040

**Published:** 2020-10-07

**Authors:** Masaki Haruna, Masaki Ogino, Toshiaki Koike-Akino

**Affiliations:** ^1^Advanced Technology R&D Center, Mitsubishi Electric Corporation (MELCO), Amagasaki, Japan; ^2^Faculty of Informatics, Kansai University, Takatsuki, Japan; ^3^Mitsubishi Electric Research Laboratories (MERL), Cambridge, MA, United States

**Keywords:** visual haptics, remote machine system, manipulation, brain wave, EEG

## Abstract

Remote machine systems have drawn a lot of attention owing to accelerations of virtual reality (VR), augmented reality (AR), and the fifth generation (5G) networks. Despite recent trends of developing autonomous systems, the realization of sophisticated dexterous hand that can fully replace human hands is considered to be decades away. It is also extremely difficult to reproduce the sensilla of complex human hands. On the other hand, it is known that humans can perceive haptic information from visual information even without any physical feedback as cross modal sensation between visual and haptics sensations or pseudo haptics. In this paper, we propose a visual haptic technology, where haptic information is visualized in more perceptual images overlaid at the contact points of a remote machine hand. The usability of the proposed visual haptics was evaluated by subject's brain waves aiming to find out a new approach for quantifying “sense of oneness.” In our proof-of-concept experiments using VR, subjects are asked to operate a virtual arm and hand presented in the VR space, and the performance of the operation with and without visual haptics information as measured with brain wave sensing. Consequently, three results were verified. Firstly, the information flow in the brain were significantly reduced with the proposed visual haptics for the whole α, β, and θ-waves by 45% across nine subjects. This result suggests that superimposing visual effects may be able to reduce the cognitive burden on the operator during the manipulation for the remote machine system. Secondly, high correlation (Pearson correlation factor of 0.795 at a *p*-value of 0.011) was verified between the subjective usability points and the brainwave measurement results. Finally, the number of the task successes across sessions were improved in the presence of overlaid visual stimulus. It implies that the visual haptics image could also facilitate operators' pre-training to get skillful at manipulating the remote machine interface more quickly.

## Introduction

Robotics have been expected to overcome the labor shortage in several countries including Japan due to population decline, declining birthrate, and aging population (The Japan Institute for Labour Policy and Training, 2016). Recent trends of autonomous system development not only in the field of automated driving technology but also leg locomotion technology have made significant progress (Feng et al., 2014; Park and Kim, 2014). Despite the progress, the realization of sophisticated dexterous hand with finger that can fully replace human hands requires decades of further advancements (SoftBank Robotics, 2017). Accordingly, a remote machine system technology has been getting attractions because of the possible operations beyond space as an alternative solution to alleviate the uneven distribution of the population.

First remote machine manipulation system was developed in the 1940s by Geortz (1952) for operating radioactive materials that were mechanically coupled from an isolated room. The concepts of “tele-existence” (Tachi et al., 1985) and telepresence (Fisher et al., 1987) showed a sense of realism and immersion to transmit the sensor information aiming to “sense of oneness” as if the remote machine was a part of his/her own limbs owing to the progress in electronics and computers since the 1980s. Those applications have become considerably important for the post coronavirus (COVID-19) society, where social distancing and tele-works are demanded. For the Fukushima nuclear disaster in 2011 following the Great East Japan Earthquake, it has been pointed out that the operation interface should be installed quickly with sufficient operator pre-training in the construction field (Asama, 2012). Virtual reality (VR) and augmented reality (AR) technologies have spread in the smartphone and game fields, and remote services with a practical deployment of the fifth generation (5G) networks in the communication field have received attraction not only by carriers but also by industries such as TOYOTA and ANA (ANA, 2018). The da Vinci Surgical System introduced in the medical field performs an operation indirectly through an operation interface on forceps in a patient's body with a capability to minimize the surgical site. Approximately 5,800 units (as of June 2020) have been sold since the company was established in 1999, and it was announced that there have been more than 2 million cases worldwide (INTUITIVE, 2019).

“Sense of Oneness,” which indicates “sense of body ownership” and/or “sense of agency,” is important for the operator who manipulates a remote machine system. Guterstam and Ehrsson (2012) reported that three elements of multisensory integration, visual consistency, and first-person perspective are required for somatosensory transfer. Although the haptic sensation presentation technology is an important “missing element” for remote machine systems, it is extremely difficult to reproduce the physical haptics of a complex human hand, and the fundamental technologies are still on the way of developing.

According to Nishio et al. (2012), a transfer of body sensation can occur when the intention of the movement and the visual feedback are reasonably sensed by the operator even if somatic sensation or haptic sensation was not present. This report suggests that the “sense of oneness” could be realized with a much simpler mechanism than existing researches. In addition, through the direct observation of the brain using inserted probe, Iriki et al. (1996) and Maravita and Iriki (2004) observed that the body ownership was acquired by short-term exercise experience in experiments where a monkey is trained to use a tool for a food. Cross-modal sensation between visual and haptics sensations have been revealing interesting phenomenon such as rubber hand illusion (RHI) (Botvinick and Cohen, 1998), full body illusion (FBI) (Lenggenhager et al., 2007), and out of body illusion (Ehrsson, 2007). Pseudo haptics (Moody et al., 2009) is also an illusion that an operator feels his/her haptic sensation stimulated only with visual sensation and without physical haptics devices. These illusions indicate the ability of high adaptation of human recognition. Moreover, with regard to vision systems, the head tracking function was greatly improved in the “VR first year” when Oculus Rift was released in 2016, and the evolution of the visual presentation technology is still in progress to achieve higher resolution, a wider field of view, and a lower price.

**Figure 1:**
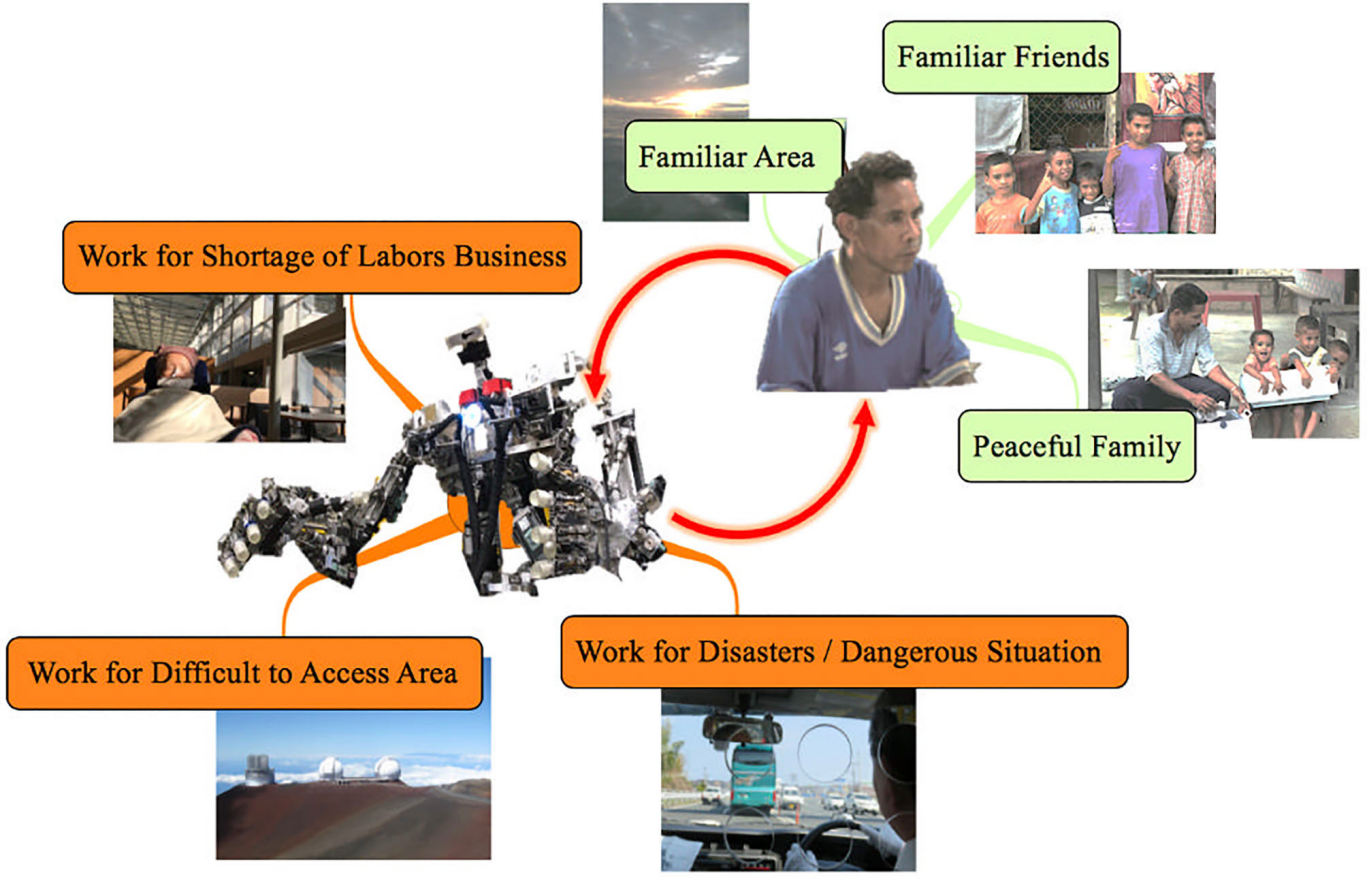
Applications by remote machine system. There are a lot of work which faces hard situation because of the shortage of labors, difficulty of access, or dangers. Remote Machine System has various possibilities to improve the situation better with promising safety for the operators, who never emigrate from their familiar area, separate from their peaceful family, nor from their familiar friends. The copyrights of all photos are permitted. The elder person of left side is the author's grand mother. The photos of the right upper side were already published in 2018 as “small world project” of which leader is the author and was available below URL. The other photos were taken by the author. https://www.mitsubishielectric.com/en/sustainability/csr/management/sdgs/pdf/Small_World_Project_201811.pdf.

In this paper, the authors propose a visual haptic technology, where haptic information is visualized in more perceptual images overlaid at the contact points of a remote machine hand. The usability of the proposed visual haptics feedback was evaluated by subject's brain waves aiming to find out a new approach for quantifying “sense of oneness.” The proposed remote machine system could be ultimately simple with a visual presentation device and a motion capture device, which is expected to solve the problem of labor shortage due to the uneven distribution of population and the problem of harsh workplace such as disaster sites. As shown in Figure 1, remote machine systems can be used not only for labor shortages, but also for a wide range of applications such as remote inspection facility maintenance, frequent natural disaster response, and telework. The contributions of our work are four-fold as follows:

- We develop a low-cost prototype system for manipulating a remote machine hand, exploiting the VR technology.
- We investigate the impact of visual haptics on teleoperation tasks via brainwave analysis.
- We demonstrate that the perceptual presence of visual haptic information overlaid in the VR headset can improve the operability.
- We propose to use a brainwave activity as an indicator to quantify the usability of the remote machine interface.

Section Existing Haptic Technology for Manipulation of Remote Machine Systems describes the existing remote machine systems and existing haptic sense presentation technologies. We show that current haptic sensation presentation devices are not at a level that can fully transfer the feeling of the remote machine to the operator and suggest that physical haptic feedback is not required for certain tasks in remote operations. Section Proposal of Visual Haptics Technology for Manipulation of Remote Machine Systems proposes the application of visual haptic technology to remote machine systems as an alternative to the existing physical haptic presentation and shows the expected effects. Section VR Experimental Environment for Evaluation of Visual Effect for Haptics shows a VR system constructed to verify the proposed hypothesis and verification experiments using brainwave measurement. Section Analysis of EEG Evoked with Visual Effect for Haptics shows the analysis results of the electroencephalogram (EEG) data in our experiments. Section Discussion gives considerations from the results obtained and shows future directions. Section Conclusion summarizes this paper with a conclusion that it was effective to transmit haptic information via visual information in remote machine manipulation. Our result is expected to contribute to the realization of a remote machine system that can perform delicate work requiring “sense of oneness” while keeping the operation interface simple and low-cost.

## Existing Haptic Technology For Manipulation of Remote Machine Systems

Multiple functions such as reaction force detection on the finger joint, shear force detection at the fingertip, and temperature detection, were studied in order to generate the haptic sensation that humans can feel naturally. In this case, the devices could be often complicated for the operator to wear on his/her hands. There are two types of devices that physically present a haptic sensation: a “grounding type” that is grounded to a fixed part and a “wearing type” that is worn on a user's hand (Table 1). The grounding type is relatively high-end; e.g., sigma.7 (produced by force dimension) can transmit haptic sensation in a space of about ϕ150 mm with 6 degrees of freedom and 1 degree of freedom for gripping status (Tobergte et al., 2011). Virtuose 6D (by haption) can transmit haptic sensation with 6 degrees of freedom in a space of about 1 m (Haption, 2019b). The wearable-type devices are easy to carry compared to the grounding-type devices because they are attached to hands, and relatively low-cost products for gaming are available on the market. As a high-end product, HGlove (by haption) can present reaction forces on three fingers (Haption, 2019a). Prime Haptic (by Manus VR) is a device that transmits contact information of each finger individually by vibration, and as it cannot provide accurate reaction force feedback, it is expected to be used in simple applications such as gaming (Manus VR, 2019a). Prime One (by Manus VR) presents haptic sensation with only one vibration attached on the back of hand (Manus VR, 2019b). HaptX Gloves is a device that realizes physical haptic feedback at multiple points. With micro-electro-mechanical systems (MEMS) fluid technology, it is designed to present a total of 120 points of pressure, i.e., 30 points on three fingers and additional 30 points on a palm, making it one of the advanced consumer devices (HaptX, 2019). Encounter-type haptic device feeds back a reaction force only when presenting contact while performing free movement; e.g., a device developed by Nakagawara et al. (2005). In addition, various researches focusing on tactile presentation were reported as a tactile display (Hayward and Cruz-Hernandez, 2000; Yamamoto et al., 2004; Wijntjes et al., 2009).

**Table 1:** Existing haptic devices for manipulation.

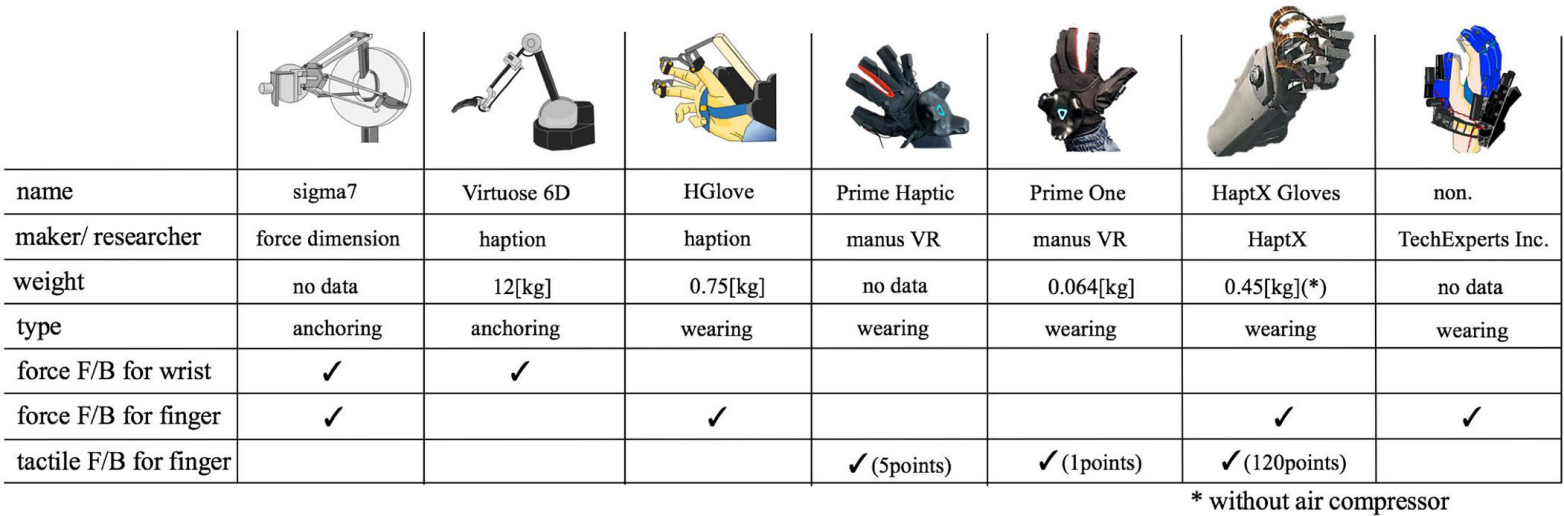

*All of the image copyrights are permitted. The three photos of “Prime Haptics,” “Prime One,” and “HaptX Gloves” were worn by the author and took by himself. The other four sketches were drawn by the author*.

The da Vinci has led the advanced researches and developments since 1980 in the context of remote machine systems. The forceps in the patient's body and the forceps operated by the surgeon are not mechanically coupled. This is the reason why da Vinci is categorized as one of remote machine systems. Despite the effectiveness of haptic presentation in various surgical support robots (King et al., 2009; Enayati et al., 2016), most surgeons found that haptic presentation was not really necessary as they could reasonably infer the sense of haptic based only on visual information when operating da Vinci, which has no haptic feedback (Kawashima, 2019). This suggests that the existing haptic sensation presentation device is not at a level that can sufficiently transmit the manipulating feeling to the operator, and that physical feedback of haptic sensation is not required for certain tasks in remote manipulation.

These facts lead that visual haptics feedback could be reasonable for remote machine systems in the aspects of simplicity and operation effects owing to the ability of high adaptation of human recognition. Pseudo haptics is not an actual physical haptics feedback but an illusion that an operator feels his/her haptic sensation stimulated only by visual sensation without physical haptics devices. Williams compared visual force feedback and physical force feedback on drill task by remote operated humanoid robot (Williams et al., 2002). It was found that visual force feedback alone can reduce the maximum force and torque magnitude by 23% compared to the case without force feedback, and that visual-only feedback outperforms physical feedback in the subjective results. Carol E. applied visual force feedback (VFF) on robot assisted surgical knots task in which information of force sensor on instrument tips were overlaid in the console image as colored circle depending on the force status (Reiley et al., 2008). It was indicated that the effects of VFF was remarkable among the surgeons, who have no robotic experience, in the performance of suture breakage rates, peak applied forces and standard deviations of applied forces. Ali compared combination of visual and physical force feedbacks on teleoperated systems (Talasaz et al., 2017). The visual force feedback improved performance in case of no physical force feedback applied and degrades in case of physical force feedback applied. It was suggested that the visual force feedback is better than the physical force feedback in the quality of knots.

## Proposal of Visual Haptics Technology For Manipulation of Remote Machine Systems

In this paper, the authors propose a visual haptic technology which visualizes haptic information in more perceptual images overlaid at the contact points of a remote machine hand. We then demonstrate the usability of the proposed visual haptics feedback by analyzing subject's brain waves to find out a potential approach for quantifying “sense of oneness.”

**Figure 2:**
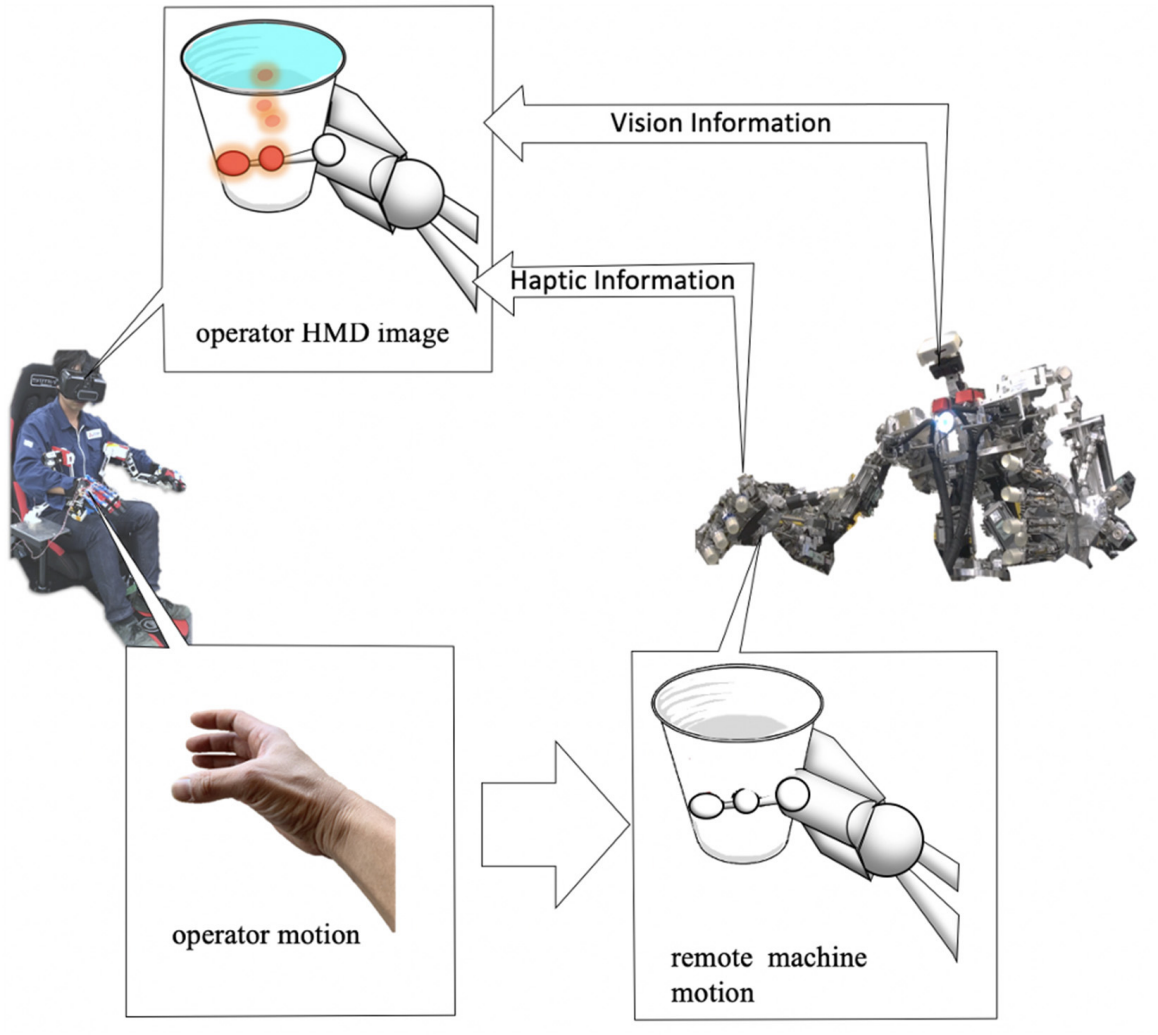
Proposed visual haptics image for manipulation. An operator controls a remote machine via a machine interface. Haptics information is measured by distributed pressure sensors of remote machine and combined with vision information via replacing the pressure information to visual expression, and the integrated image is transmitted to the operator. The operator separates the integrated vision information to vision information and haptic information intuitively. This proposal is based on the fact that most surgeons found that haptic presentation was not really necessary as they could reasonably infer the sense of haptic based only on visual information, and that recognition of haptics information is owing to not only haptics sense but also vision sense.

**Figure 3:**
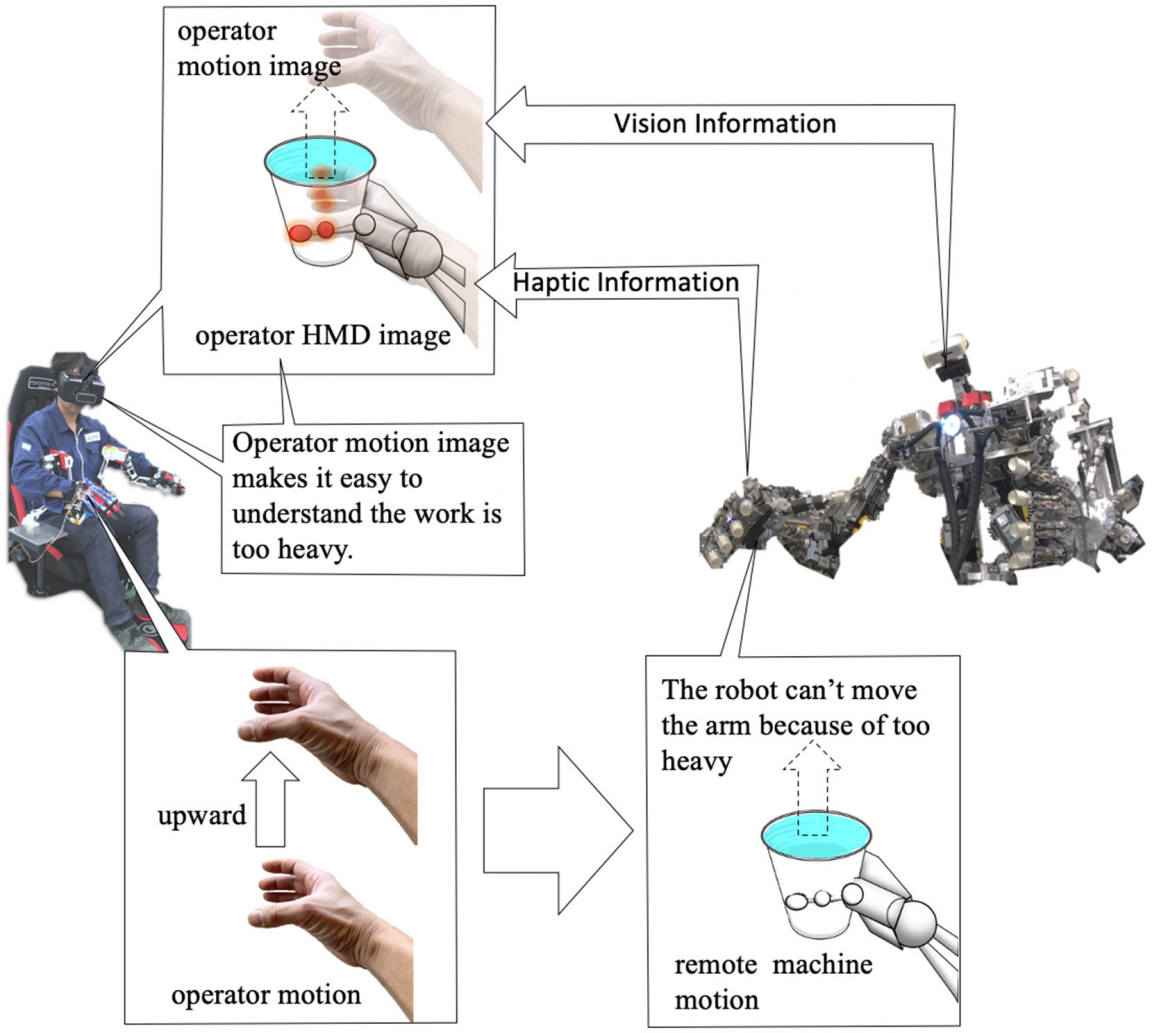
The other advantage of proposed visual haptic image for manipulation. An operator controls a remote machine via a machine interface. Haptics information is measured by distributed pressure sensors of remote machine and combined with vision information via replacing the pressure information to visual expression, and the integrated image is transmitted to the operator. Moreover, the operator motion is superimposed on the integrated image. The level of harmony between the remote machine motion and the superimposed operator motion makes the operator feel effect of gravity acting on the grasping object.

In remote control, there is much haptic information obtained from vision. In particular, a person can infer from the deformation, texture or shadow of an object due to contact with a hand. However, in the case of a highly brittle object with raw visual information alone, the object may be accidentally damaged. For hard objects, there is a potential risk that the machine hand is instead damaged. In order to deal with these problems, we propose a new remote machine interface adopting visual haptics sensation that visually presents physical haptic information in an overlay image at the contact points of the mechanical hand (Figure 2). Although there are some related work that haptic information are displayed in the form of numerical information or vectors, there were no prototypes that visualize haptic information in more perceptual images overlaid at the contact points of a real robot hand for remote machine manipulation systems to date when we filed the corresponding patent in 2016 to the best of authors' knowledge. The method using numerical or vector expressions of the haptic information were implemented to our remote machine system and found a major drawback experimentally that it will impose higher task loads for the operator to recognize the situation of the robot grasping under the constrained field of view. Our proposed method has a distinguished advantage in alleviating such issues. In addition to presenting haptic information for a visible part of the machine hand, it is also possible to project an overlay image when the machine hand is occluded in the work area by obstacles (Figure 2). Such a situation happens frequently for regular object gripping tasks. Hence, superimposing pseudo haptic information on remote machine images exploiting AR technologies will be of great advantage in practice. It is also possible to superimpose a computer graphic (CG) video of remote machine or human hand on the remote machine video captured at remote sites. In our system using the AR technology, the operator can readily confirm whether the remote robot is properly following the intended motion of the operator or is failed due to overload or some troubles (Figure 3). Accordingly, the application of AR and VR technologies can considerably facilitate remote control without relying on any complicated device platforms. In fact, our proof-of concept system prototyped in this work was realized with a simple configuration equipped with just two types of interfaces; a visual display device and a motion capture interface.

Brainwave measurement and analysis are applied to quantify the usability or feeing of the proposed visual haptics feedback. In the area of pseudo haptics and remote machine operations, there is no precedent researches so far. The correlation between subjective results, task performance, and the brainwaves data are investigated in this paper.

## VR Experimental Environment for Evaluation of Visual Effect For Haptics

In this section, we describe a VR experiment system to verify our hypothesis that visual haptics can facilitate manipulation tasks for remote machine systems. Two cases are considered for this verification experiment. The first experiment is denoted as “scene-A,” which is a manipulation task without visual haptics feedback but with raw visual perception images. The second experiment is called “scene-B,” which is an identical manipulation task except that both visual information and visual haptics information are superimposed. The objective of these experiments is to analyze usability of interface with and without visual haptics feedback through the measurement of task performance and brainwaves for multiple different subjects.

**Figure 4:**
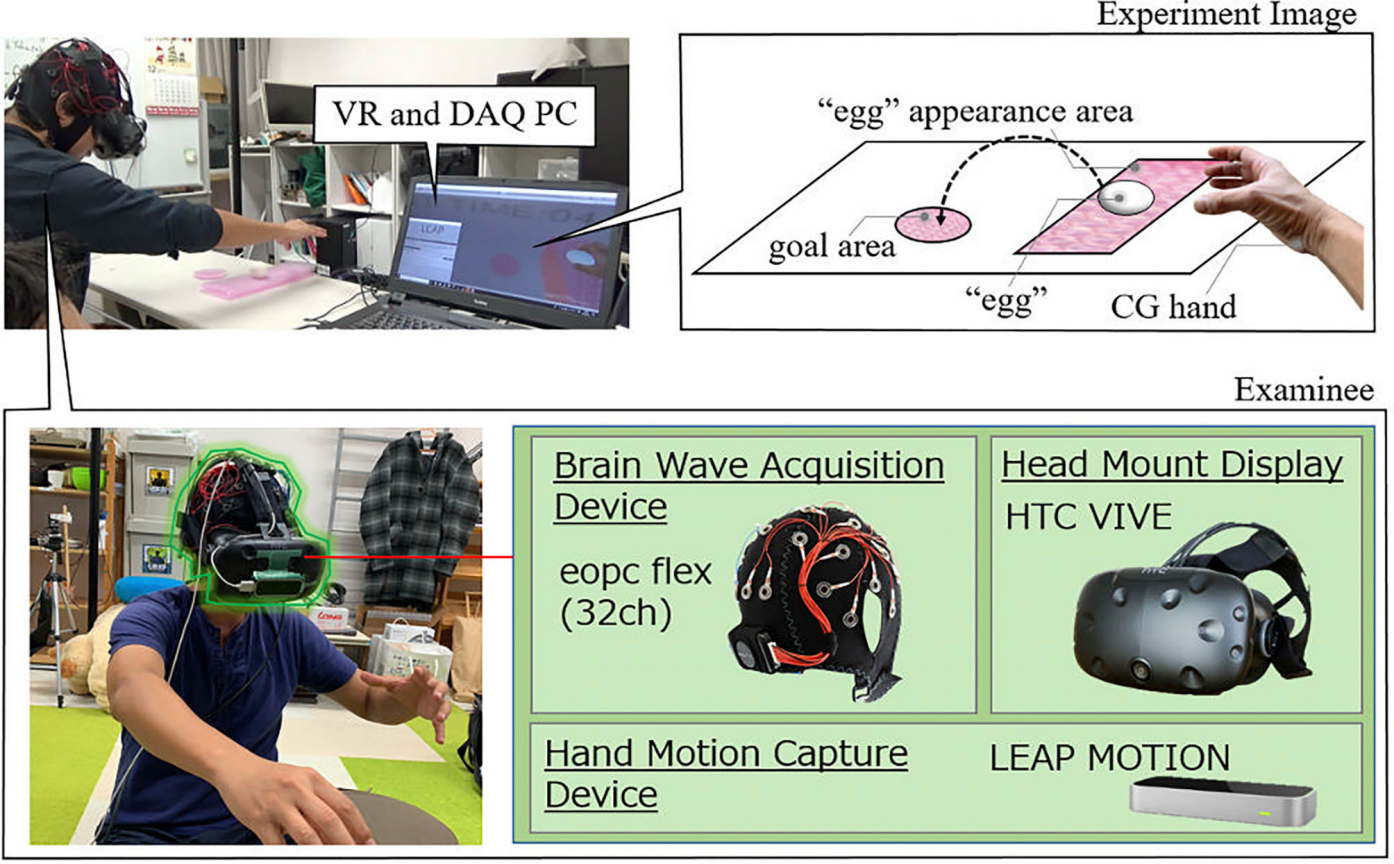
VR experimental environment. The developed VR experimental environment consists of a head mount display for vision image transmission to an operator (a subject), a hand motion capture device for detecting hand and finger motion, and brain wave acquisition device for measuring the brain wave during evaluation experiments. The evaluation task is designed to avoid the getting used to manipulate an object over repeated sessions in order to evaluate the effect of the proposed visual haptics. During the experiments, an egg appeared with random orientation every time makes it difficult for a subject to learn the hand shape which can grasp the appeared egg.

**Figure 5:**
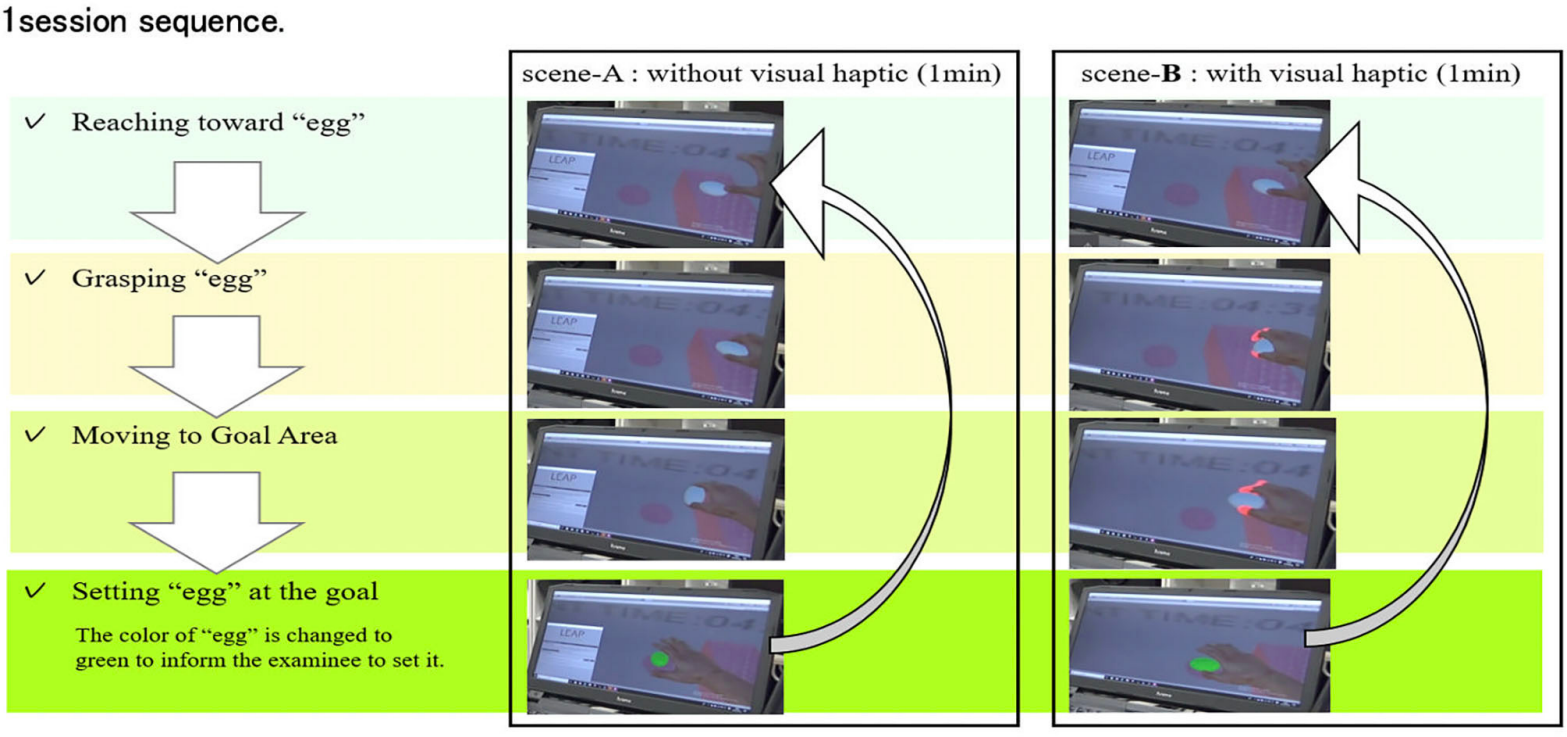
1session sequence. A subject wearing head-mounted display (HMD) is instructed to perform sequential tasks of relocating virtual “eggs” of random size appearing at a random location in a predetermined “appearance range” on the right side in the VR space to a “goal area” in the center region (Figure 4). During this experiment, reaching, grasping, moving and releasing target eggs are performed continuously and repeatedly for about 1 min for each one session of scene-A (without visual haptics) and scene-B (with visual haptics). These subjects are instructed to grasp the egg gently not to break and to move many eggs to the goal as possible as they can. In this experiment, the egg is not modeled to break when the excessive force is added.

The subjects wear a head-mounted display (HMD), in front of which a hand capture device is equipped with. At the same time, the subjects also wear a 32-channel electroencephalogram (EEG) sensor, which measures brainwaves of the user during the experiments. Specifically, the devices used in our experiments include Vive (HTC) for the HMD, Leap Motion (Leap) for the hand capture device, EPOC Flex (Emotiv) for the EEG sensor, a graphic processing unit (GPU)-equipped notebook computer for VR experiment and data acquisitions, and unity 3D (Unity Technologies) for VR environment design. The subject was asked to perform sequential tasks of relocating virtual “eggs” of random size appearing at a random location in a predetermined “appearance range” on the right side in the VR space to a “goal area” in the center region (Figure 4). During this experiment, multi-function operations including reaching, grasping, moving and releasing target eggs are performed continuously and repeatedly for about 1 min in one session (Figure 5). The virtual CG hand appearance in the VR space was designed to be similar to a real human hand. This is because we intended to focus only on the effect of visual haptics information by excluding a concern that a sense of discomfort induced by a different appearance affects the evaluation for the case when a mechanical hand is visualized. As a haptic information, we used a visual feedback by overlaying translucent red images on a CG hand fingertip in the VR space only at a time when the hand is in contact with an object. In this experiment, the subject is notified of the successful arrival at the goal area by changing the color of eggs from white to green. The data measured in the experiment for each subject are six sets of EEG measurement data consisting of three sessions without visual haptic effect (scene-A) and three sessions with visual haptic effect (scene-B), one set of video to record a series of one subject, and one subjective questionnaire for usability evaluation.

**Figure 6:**
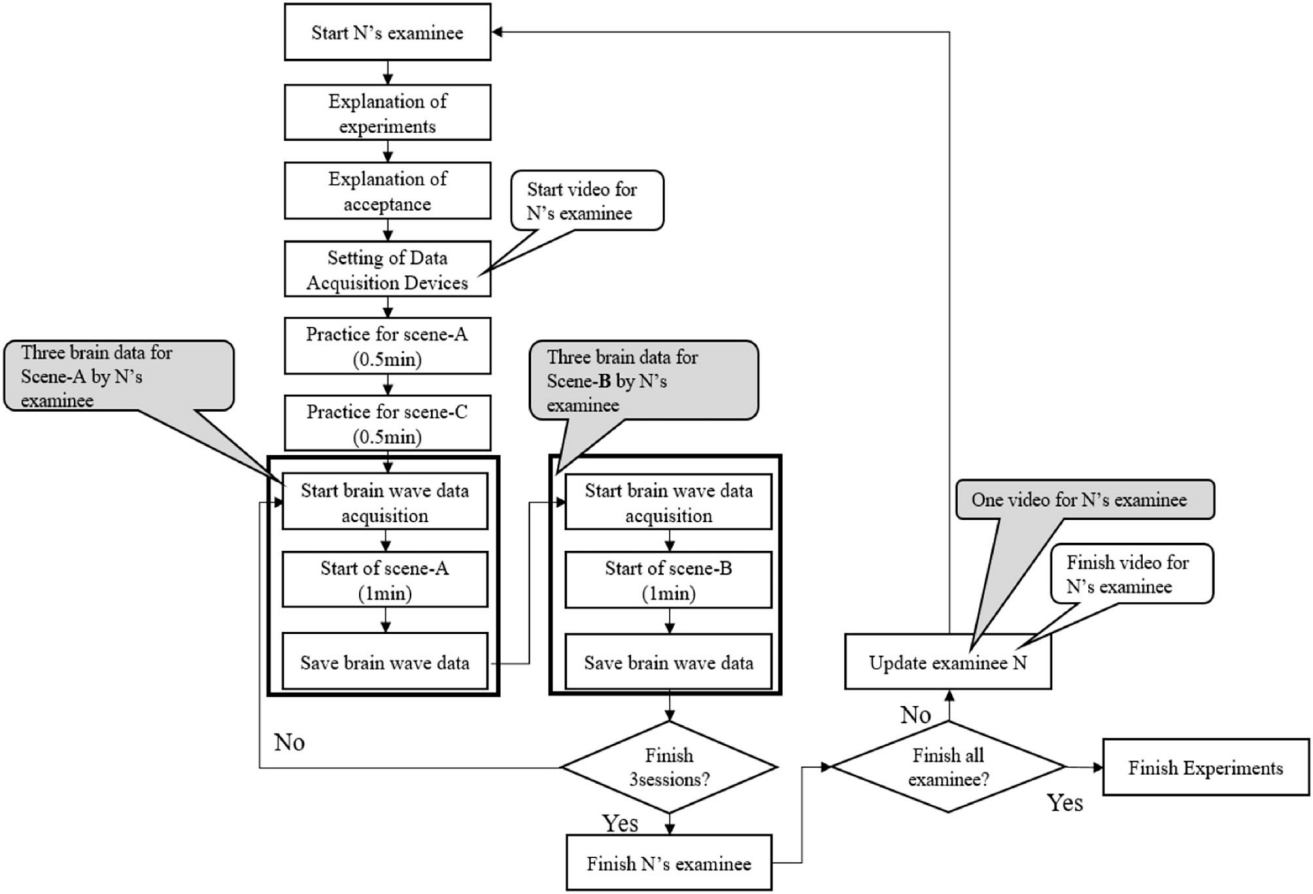
Experimental sequence.

The experiment sequence is shown in Figure 6. After explaining the experiment and the consent document, the above-mentioned set of devices is worn to each subject. The scene-A and scene-B are performed alternately for three sessions, respectively, after 0.5 min period practice, respectively. This sequence was conducted for 12 different subjects who have no particular physiological disabilities. These subjects are instructed to grasp the egg gently not to break and to move many eggs to the goal as possible as they can. In this experiment, the egg is not modeled to break when the excessive force is added.

**Figure 7:**
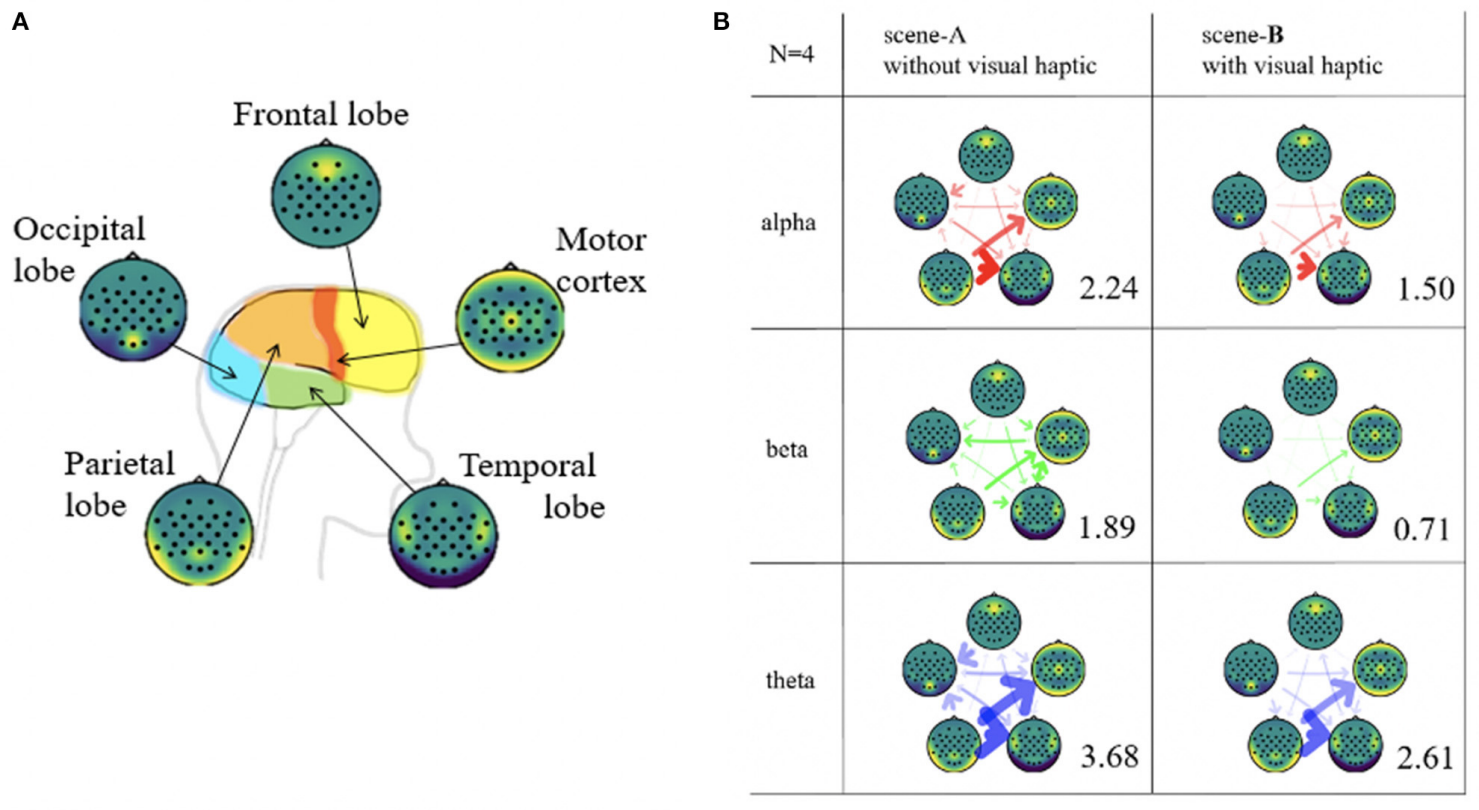
Results of brain wave analysis by SCoT. **(A)** shows the spatial arrangement of the brain corresponding to five parts classified by SCoT analysis. The arrows in **(B)** indicate the direction of the information flow in the brain (the time sequence in which it is excited). The thickness of the arrow line represents the amount of information flow. A numerical value to denote the total amount of information flows is also present.

The brainwave data acquired by the EEG sensor are visualized by analyzing the cognitive information flow in the brain through the use of smooth coherence transform (SCoT) library (Billinger et al., 2014). While the original 32-ch electrodes are spatially mapped to cover most major scalp locations specified in the standard international 10–20 system, we selected five source locations projected on the frontal, occipital, parietal, temporal, and motor regions for analyzing the information flows as shown in Figure 7A. The time-series data at a sampling rate of 128 Hz for about 1 min is divided into short-time sequences of 0.5 s, and the information flow in the brain is analyzed using stationary vector autoregressive (VAR) model of the 30th order, which is sufficient to pass a statistical whiteness test. We use full frequency directed transfer function (ffDTF) for the causality metric to analyze the information flow (Korzeniewska et al., 2003). We preprocessed the EEG data by excluding measurements of three subjects for all sessions and one subject for last two sessions whose sensor sensitivity score was below 50%. Figure 7B shows one of the visualization results as an example. The information flow results of three sessions for each subject were evaluated for three frequency bands of α, β, and θ waves. The arrows in the figure indicates the direction of the information flow in the brain (the time sequence in which it is excited). The thickness of the arrow line represents the amount of information flow. A numerical value to denote the total amount of information flows is also present in Figure 7B.

## Analysis of EEG Evoked With Visual Effect for Haptics

**Figure 8:**
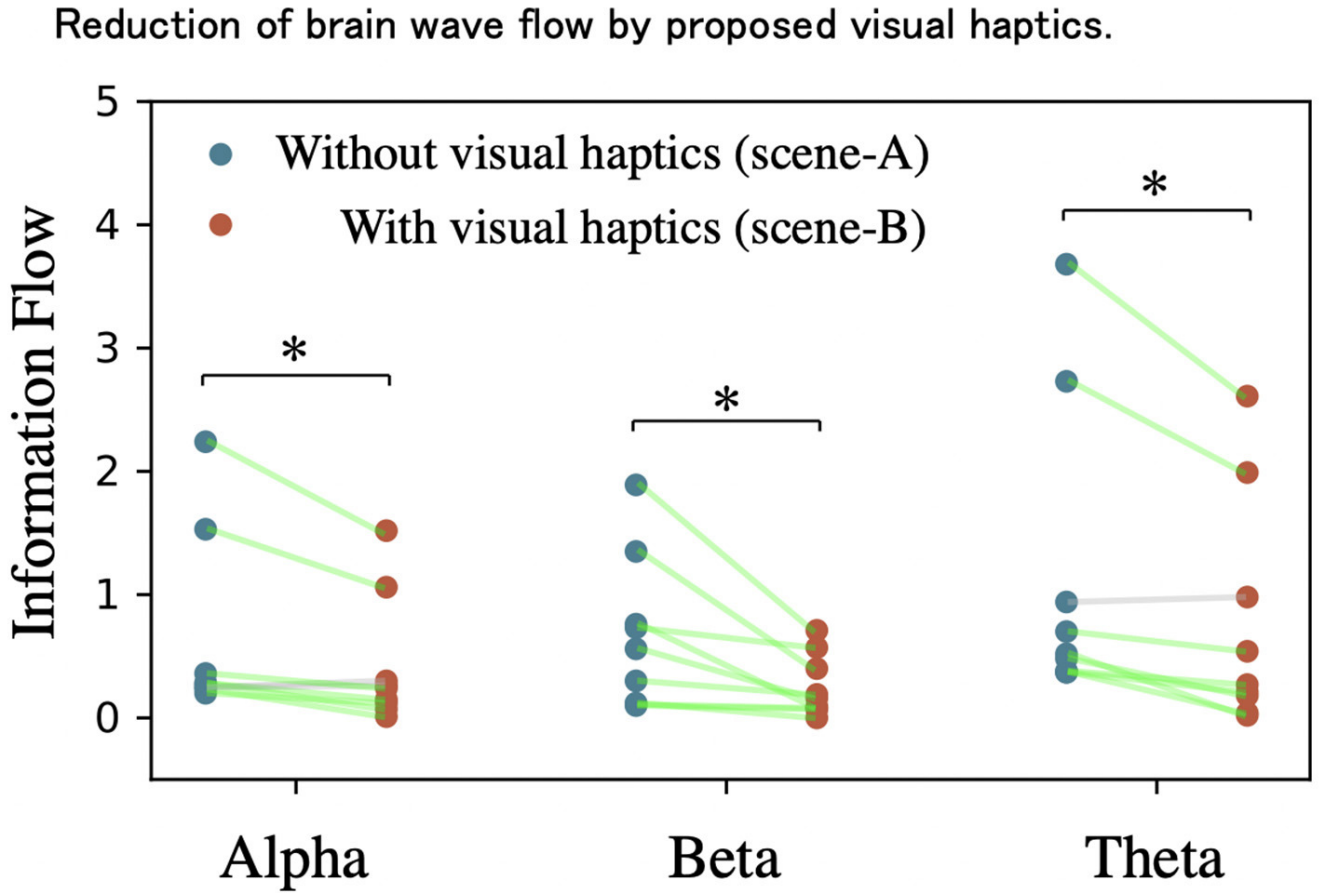
Reduction of brain wave flow by proposed visual haptics. The vertical axis shows the reduction of the information flow in the brain in in each remaining nine subjects for three frequency bands from the experiments without visual haptics to with visual haptics. Through all of the bands, the reduction ratios were verified for the whole α-wave (*t*_9_ = 2.557, *p* = 0.034 < 0.05, paired *t*-test), β-wave (*t*_9_ = 2.831, *p* = 0.022 < 0.05, paired *t*-test), and θ-wave (*t*_9_ = 3.181, *p* = 0.013 < 0.05, paired *t*-test). *indicates significant difference (*p* < 0.05) in paired *t*-test.

Figure 8 depicts the change of the information flow in the brain of the remaining nine subjects for three frequency bands between the experiments with/without visual haptics. The amount of the information flow in scene-B is significantly reduced compared to scene-A for the whole α-wave (*t*_9_ = 2.557, *p* = 0.034 < 0.05, paired *t*-test), β-wave (*t*_9_ = 2.831, *p* = 0.022 < 0.05, paired *t*-test), and θ-wave (*t*_9_ = 3.181, *p* = 0.013 < 0.05, paired *t*-test). The average of the information flow reduction rates by visual haptics image across all subjects was 45%. This result could be because the operators require to compensate for the lack of haptic information in the VR space by using indirect clues and experiences. This result suggests that superimposing visual effects may be able to reduce the cognitive burden on the operator during the manipulation for the remote machine system.

**Figure 9:**
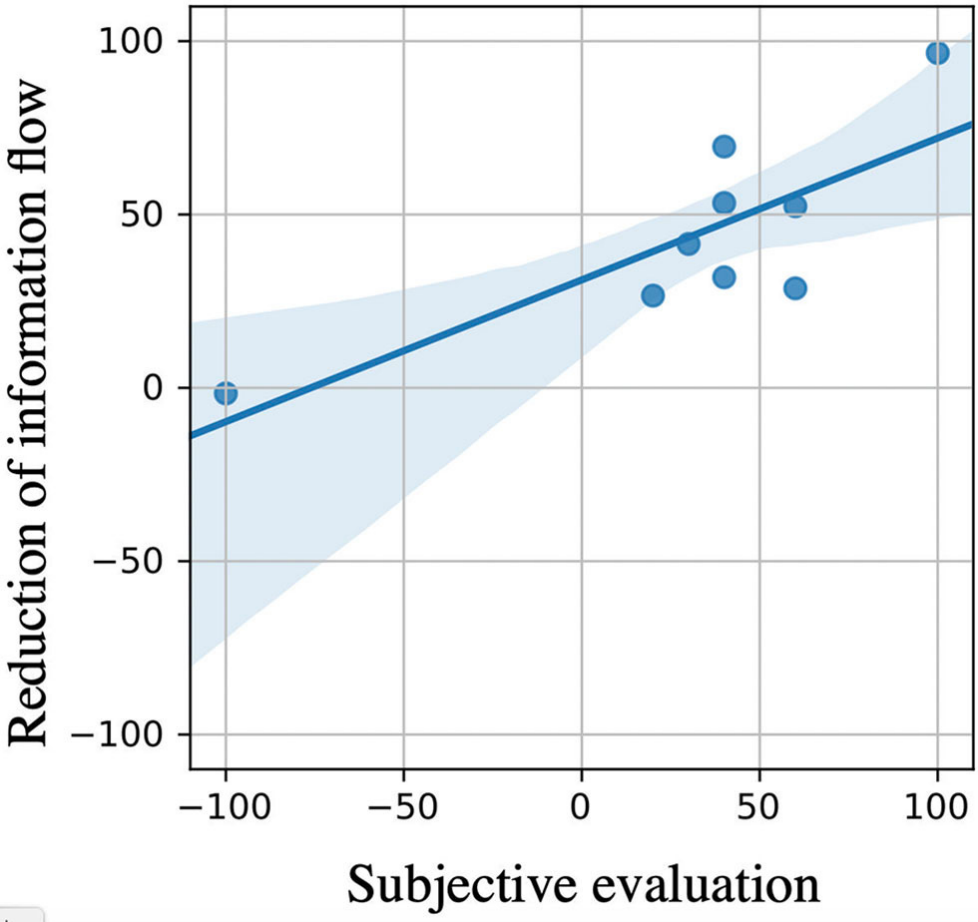
Relationship between brain wave results and feeling points of subjects. This figure shows the correlation between the reduction of the information flow in the brain by visual haptics image described in the vertical axis and the “subjective evaluation” in the horizontal axis which were answered by each subject in the post questionnaire of the experiment (+100 corresponds to “highly effective,” 0 corresponds to “no effective at all,” and – “100 corresponds to disturbed usability by visual haptics”). Therefore, the “subjective evaluation” is positive if the subject feels the advantage of the visual haptics feedback and negative if the subject feels the disadvantage. This result shows a high correlation, i.e., Pearson correlation factor of 0.795 (*p*-value: 0.011 < 0.05), between the subjective usability points and the brainwave measurement results. The shaded area is the 90% confidence interval.

Figure 9 shows the correlation between the reduction ratio of the information flow in the brain by visual haptics image described in the vertical axis and the “subjective evaluation” in the horizontal axis which were answered by each subject in the post questionnaire of the experiment.

The shaded area is the 90% confidence interval. The “subjective evaluation” is positive if the subject feels the advantage of the visual haptics feedback, and negative if the subject feels the disadvantage. This result shows a high correlation, i.e., Pearson correlation factor of 0.795 (*p*-value: 0.011 < 0.05), between the subjective usability points and the brainwave measurement results.

**Figure 10:**
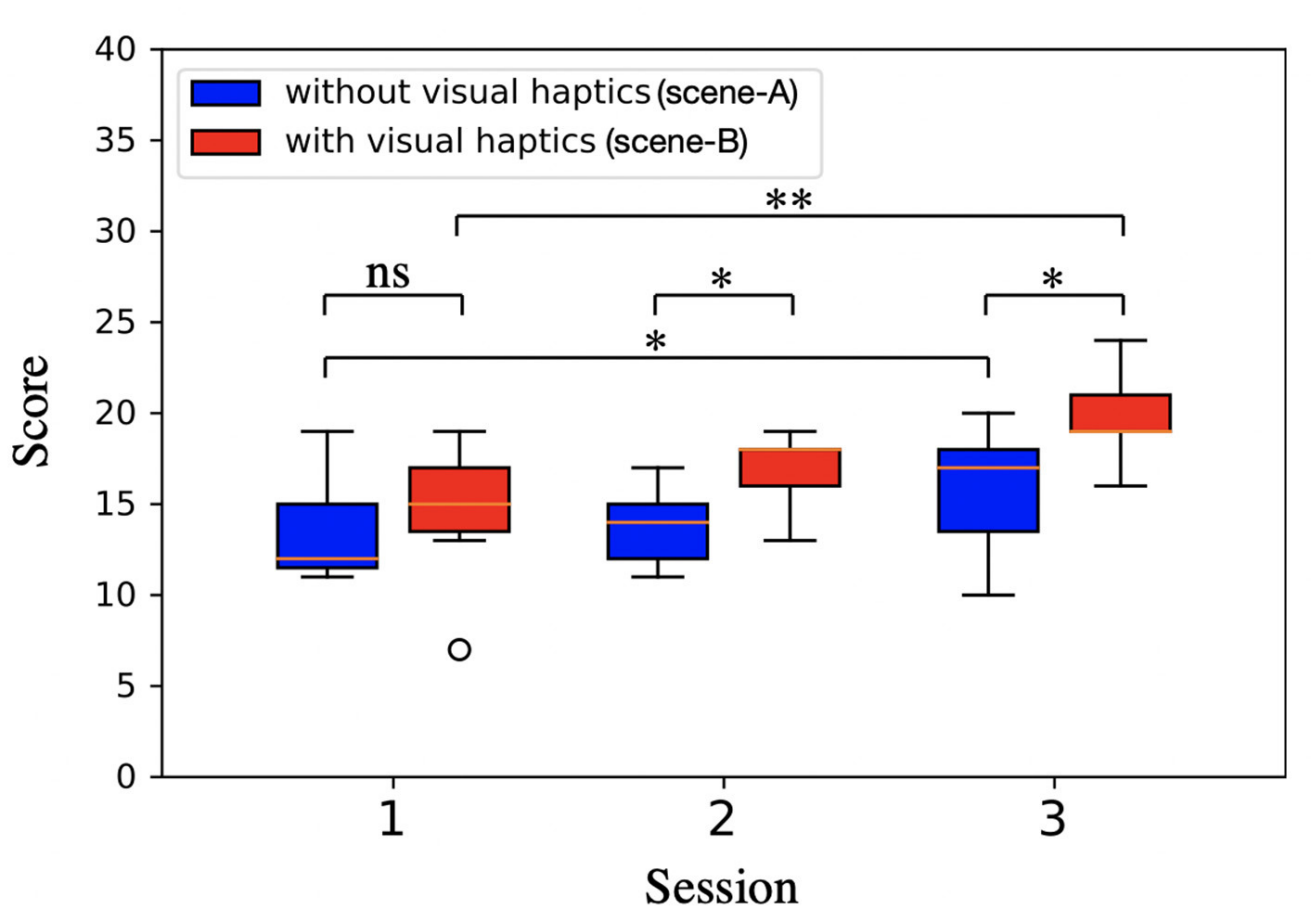
Contribution of visual haptics for operation learning. From the video data recorded for each subject, we measured the task performance in “score” expressed in the vertical axis, which is defined as the number of trial times that the target eggs were successfully transported to the goal area subtracted by the number of times that eggs were dropped or failed to grip The “score” constantly improves both in scene-A (without visual haptics image) and in scene-B (with visual haptics image). According to the statistical analysis, the “score” improvements are more between 1st and 3rd session in scene-B (*t*_7_ = −5.755, *p* = 0.001 < 0.01, paired *t*-test) rather than in scene-A (*t*_7_ = −2.905, *p* = 0.027 < 0.05, paired *t*-test). It implies that the visual haptics image could contribute to effective familiarity development which is practically useful for the operators to learn the remote machine interface quickly in pre-training sessions. *indicates significant difference (*p* < 0.05) in paired *t*-test. **indicates significant difference (*p* < 0.01) in paired *t*-test.

In addition, we measured the task performance in “score,” which is defined as the number of trial times that the target eggs were successfully transported to the goal area subtracted by the number of times that eggs were dropped or failed to grip. Figure 10 shows the result of counted score as a function of session. The results of seven subjects are shown, excluding the result of two subjects. The one subject's EEG data was available only for the 1st session because the sensor sensitivity score for last two session was below 50%. The other subject was not consistently incentivized to conduct the tasks in terms of maximizing successful trials or minimizing failures and just concerned smooth transportation without carrying the number of success, and mentioned that he strongly took care not to break the egg and forgot the requirement to get high score especially in case of scene-B because he could check the grasp status using the information of visual haptics feedback. The “score” constantly improves both in scene-A (without visual haptics image) and in scene-B (with visual haptics image). According to the statistical analysis, the “score” improvements are more between 1st and 3rd session in scene-B (*t*_7_ = −5.755, *p* = 0.001 < 0.01, paired *t*-test) rather than in scene-A (*t*_7_ = −2.905, *p* = 0.027 < 0.05, paired *t*-test). It implies that the visual haptics image could contribute to effective familiarity development which is practically useful for the operators to learn the remote machine interface quickly in pre-training sessions.

## Discussion

Through VR experiments and the statistical analysis of the brainwave by EEG, the subjective evaluation and the “score” of the task, the proposed visual haptics image overlaid on the contact points were verified at the three points: (1) the reduction of the information flow in the brain with visual haptics, (2) the high correlation between the subjective usability points and the brainwave measurement results, and (3) the better improvement of the “score” with visual haptics. In addition, some potential effects and improvement points were found to reveal adequate hand sensations including cross modal for a remote machine system.

**Figure 11:**
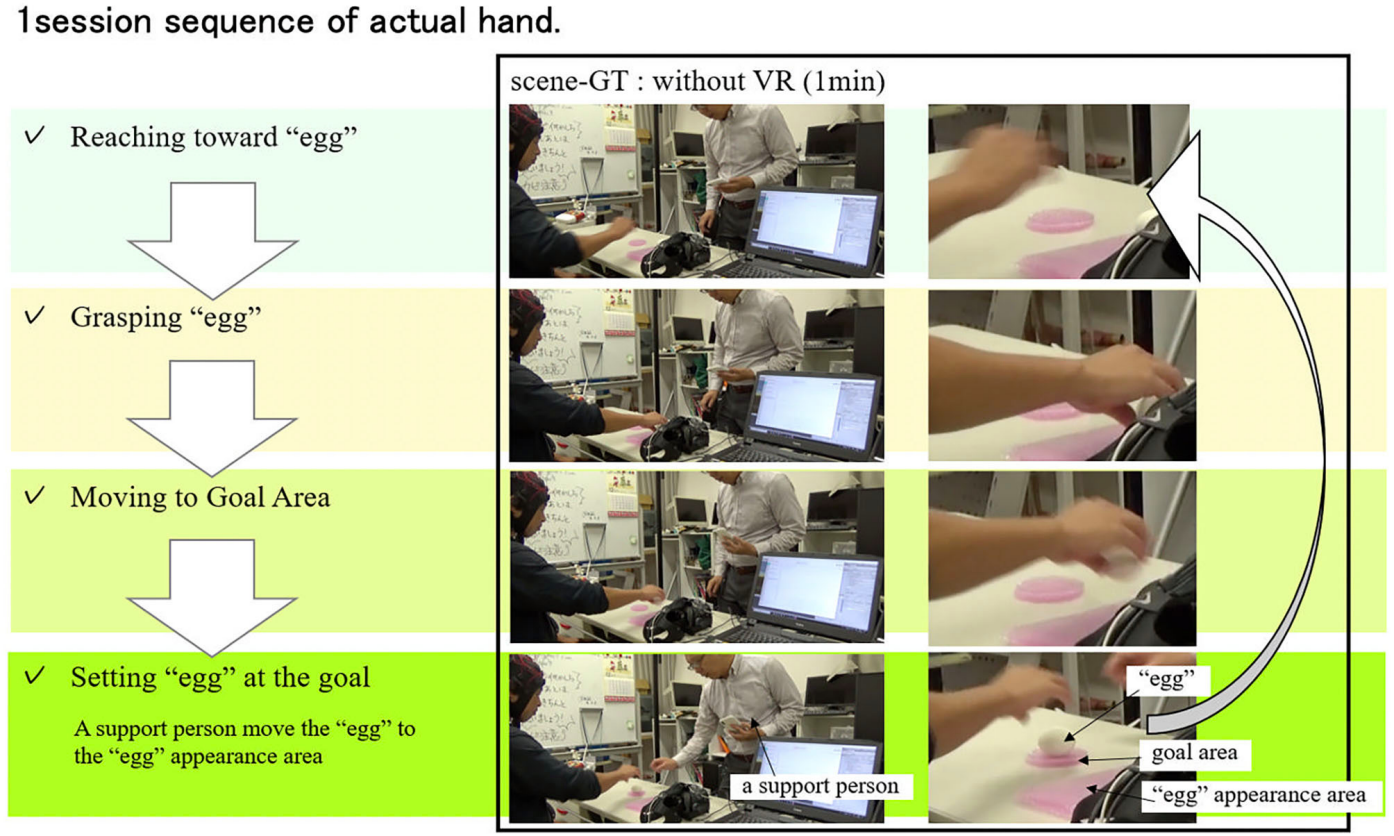
1session sequence of actual hand. The persons in these photos are the authors. This figure shows the experiment using his own hand. In this case, the amount of information flow in the brain was 1.6 as the average of all the measurements. In the VR space without visual haptics image the amount of information flow in the brain was 1.2. In the VR space with visual haptics image, the amount of information flow in the brain was 0.7. As one of potential reasons, the real world has even more amount of perceptual information than the VR space for subjects to recognize. This could indicate the difficulties of easily influenced by all inputs from outside and inside of the subject. This kind of experiments should be designed carefully for the other multi modal experiments and meaning of information flow in brain could be deepened through various evaluation experiments.

As shown in EEG analysis of Figure 9, the cognitive change in the amount of information flow in the brain was highly correlated with the improvement in operability. This motivates us to consider a future interface design measuring the brainwave information flow as a quantitative index to estimate the operability on the fly. For example, it would be possible to use the information flow in the brain to evaluate the “familiarity” in place of the direct task score used in Figure 10 (number of times the eggs were successfully transported) in this verification experiment. While our experiments were based on virtual hand in the VR environment without any real haptic sensation, we could consider a similar task using real hands of subjects. Figure 11 shows the experiment using his own hand. Similar to the VR experiment, the subject is asked to grab a real egg with his right hand, transport it to a “goal area,” and release it. The relocated egg was manually returned to the original “egg” appearance area by a support person. In this case, the amount of information flow in the brain was 1.6 as the average across all the measurements, whereas it was 0.7 and 1.2 in the VR space with and without visual haptics, respectively. As one of potential reasons, the real world has even more amount of perceptual information than the VR space for subjects to recognize. Hence, while we could interpret the amount of the information flow as the usability measure as shown in Figure 9 for our experiments, more practical multi-modal experiments should be designed carefully to validate the methodology based on the brainwave. Our experiment used a specific target object whose shape is egg-like elliptic so that the subjects do not learn the gripping condition as a hand shape by rolling around. We should evaluate more rigorous scenarios where the object shape can vary in a repetitive task. In the future work as one of the explorations, we plan to evaluate the case of the similar VR space synchronized with the real-world eggs so that the users can sense a real haptic information when touching the VR object.

The effectiveness of the proposed visual haptics image overlaid at the contact points was verified as shown in Figures 8, 9 because the visual haptics image was useful to understand the grasping status intuitively without the additional eye movement as in case of visual information displayed in the form of numerical information or vectors. It was demonstrated that the proposed visual haptics can complement the haptics information intentionally which are missing in the remote machine systems. In case of one of the subjects who was visual artist and answered the visual haptics image made interruption to grab the egg, the reduction of the information flow in the brain in Figure 9 was almost zero and the subjective evaluation was −100. These facts are also interesting in the potential indication of utilizing information flow in brain for evaluation of user's psychologic feelings. Nonetheless, it has not yet been verified whether our visual feedback was used for haptic information or just for a trigger of lifting. Essentially, we leave a future work to explore how the human uses haptics for grasping task. In order to properly evaluate the familiarity with the information flow in the brain, we shall consider more follow-up experiments with a task setting in which multiple different operations are analyzed independently such as reaching, grasping, transporting, and releasing under a consistent score setting. For the next step, we plan to examine the relative evaluation with modals other than visual information; e.g., the method of presenting contact with haptic information such as auditory information and vibration.

## Conclusion

Practical development of remote machine systems are of great importance to deal with various society issues such as the labor shortages in several countries in particular for post-COVID-19 era. However, it is extremely difficult to reproduce the sensilla of complex human hands. In this paper, the authors proposed a visual haptic technology to invoke haptic sensation by visual stimulus with perceptual images overlaid at the contact points of the remote machine hand. We evaluated the usability of the proposed visual haptics through the analysis of subject's brain waves to find out a new methodology for quantifying “sense of oneness.” Proof-of-concept experiments using VR were executed to verify three outcomes. Firstly, the information flow in the brain were significantly reduced with the proposed visual haptics for the whole α, β, and θ-waves by 45% across nine subjects. This result suggests that superimposing visual effects may be able to reduce the cognitive burden on the operator during the manipulation for the remote machine system. Secondly, high correlation (Pearson correlation factor of 0.795 at a *p*-value of 0.011) was verified between the subjective usability points and the brainwave measurement results. Finally, the number of the task successes over session repetition were improved with visual haptic feedback. It implies that the visual haptics image could contribute to effective familiarity development which is practically useful for the operators to learn the remote machine interface quickly in pre-training sessions.

## Data Availability Statement

The datasets generated for this study are available on request to the corresponding author.

## Ethics Statement

The studies involving human participants were reviewed and approved by the ethics committee of Kansai University as HR2019-13. The patients/participants provided their written informed consent to participate in this study. Written informed consent was obtained from the individual(s) for the publication of any potentially identifiable images or data included in this article.

## Author Contributions

TK-A contributed the analysis of the brain wave. MO contributed the development of the verification experiment and analysis of the brain wave. MH proposed the hypothesis of visual haptics, designed the evaluation method, development of the verification experiment and analysis of the brainwave and correcting the data from the subjects. All authors contributed to the article and approved the submitted version.

## Conflict of Interest

MH was employed by the company Mitsubishi Electric Corporation. TK-A was employed by Mitsubishi Electric Research Laboratories. The remaining author declares that the research was conducted in the absence of any commercial or financial relationships that could be construed as a potential conflict of interest.

## References

[B1] ANA (2018). AVATAR XRPIZE. Available online at: https://avatar.xprize.org/prizes/avatar (accessed August 24, 2020).

[B2] Asama H. (2012). Remote control technology for response of nuclear power plant accidents. J. Robot. Soc. Japan 30, 588–591. 10.7210/jrsj.30.588

[B3] Billinger M. Brunner C. Müller-Putz G. R. (2014). SCoT: A python toolbox for EEG source connectivity. Front. Neuroinform. 8:22. 10.3389/fninf.2014.00022 24653694 PMC3949292

[B4] Botvinick M. Cohen J. (1998). Rubber hands “feel” touch that eyes see. Nature 391:756. 10.1038/35784 9486643

[B5] Ehrsson H. H. (2007). The experimental induction of out-of-body experiences. Science 317:1048. 10.1126/science.1142175 17717177

[B6] Enayati N. De Momi E. Ferrigno G. (2016). Haptics in robot-assisted surgery: challenges and benefits. IEEE Rev. Biomed. Eng. 9, 49–65. 10.1109/RBME.2016.2538080 26960228

[B7] Feng S. Whitman E. Xinjilefu X. Atkeson C. G. (2014). “Optimization based full body control for the atlas robot,” in IEEE-RAS International Conference on Humanoid Robots (Madrid: IEEE), 120–127. 10.1109/HUMANOIDS.2014.7041347

[B8] Fisher S. S. McGreevy M. Humphries J. Robinett W. (1987). “Virtual environment display system,” in Proceedings of the 1986 Workshop on Interactive 3D Graphics, I3D 1986 (Chapel Hill, NC). 10.1145/319120.319127

[B9] Geortz R. C. (1952). Fundamentals of general-purpose remote manipulators. Nucleonics 10, 36–42.

[B10] Guterstam A. Ehrsson H. H. (2012). Disowning one's seen real body during an out-of-body illusion. Conscious. Cogn. 21, 1037–42. 10.1016/j.concog.2012.01.018 22377139

[B11] Haption S.A. (2019a). HGLOVE. Available online at: https://downloads.haption.com/marketing/datasheet/Datasheet-HGlove-2019.pdf (accessed August 24, 2020).

[B12] Haption S.A. (2019b). VIRTUOSE 6D. Available online at: https://downloads.haption.com/marketing/datasheet/Datasheet-Virtuose6D-2019.pdf (accessed August 24, 2020).

[B13] HaptX (2019). HaptX/Glove. Available online at: https://haptx.com/technology/ (accessed August 24, 2020).

[B14] Hayward V. Cruz-Hernandez J. M. (2000). “Tactile display device using distributed lateral skin stretch,” in Proceedings of the Haptic Interfaces for Virtual Environment and Teleoperator Systems Symposium, ASME International Mechanical Engineering Congress & Exposition 2000 (Orlando, FL), 1309–1314.

[B15] INTUITIVE (2019). INTUITIVE Announces Second Quarter Earnings. Available online at: https://isrg.intuitive.com/news-releases/news-release-details/intuitive-announces-second-quarter-earnings/ (accessed August 24, 2020).

[B16] Iriki A. Tanaka M. Iwamura Y. (1996). Coding of modified body schema during tool use by macaque postcentral neurones. Neuroreport 7, 2325–30. 10.1097/00001756-199610020-00010 8951846

[B17] Kawashima K. (2019). Haptics in surgical robot for minimally invasive surgery. J. Robot. Soc. Japan 37, 405–408. 10.7210/jrsj.37.405

[B18] King C. H. Culjat M. O. Franco M. L. Lewis C. E. Dutson E. P. Grundfest W. S.. (2009). Tactile feedback induces reduced grasping force in robot-assisted surgery. IEEE Trans. Haptics 2, 103–110. 10.1109/TOH.2009.4 27788101

[B19] Korzeniewska A. Mańczak M. Kamiński M. Blinowska K. J. Kasicki S. (2003). Determination of information flow direction among brain structures by a modified directed transfer function (dDTF) method. J. Neurosci. Methods. 125, 195–207. 10.1016/S0165-0270(03)00052-9 12763246

[B20] Lenggenhager B. Tadi T. Metzinger T. Blanke O. (2007). Video ergo sum: Manipulating bodily self-consciousness. Science 317, 1096–9. 10.1126/science.1143439 17717189

[B21] Manus VR (2019a). Prime Haptic. Available online at: https://manus-vr.com/prime-haptic-gloves/ (accessed June 19, 2019).

[B22] Manus VR (2019b). Prime One. Available online at: https://manus-vr.com/prime-haptic-gloves/ (accessed June 19, 2019).

[B23] Maravita A. Iriki A. (2004). Tools for the body (schema). Trends Cogn. Sci. 8, 79–86. 10.1016/j.tics.2003.12.008 15588812

[B24] Moody L. Waterworth A. Arthur J. G. McCarthy A. D. Harley P. J. Smallwood R. H. (2009). Beyond the visuals: Tactile augmentation and sensory enhancement in an arthroscopy simulator. Virtual Real. 13, 59–68. 10.1007/s10055-008-0106-x

[B25] Nakagawara S. Kajimoto H. Kawakami N. Tachi S. Kawabuchi I. (2005). “An encounter-type multi-fingered master hand using circuitous joints,” in Proceedings - IEEE International Conference on Robotics and Automation (Barcelona), 2667–2672. 10.1109/ROBOT.2005.1570516

[B26] Nishio S. Watanabe T. Ogawa K. Ishiguro H. (2012). “Body ownership transfer to teleoperated android robot,” in Social Robotics. ICSR 2012. Lecture Notes in Computer Science, Vol. 7621, eds S. S. Ges, O. Khatib, J. Cabibihan, R. Simmons, and M. Williams (Berlin; Heidelberg: Springer), 398–407. 10.1007/978-3-642-34103-8_40

[B27] Park H.-W. Kim S. (2014). The MIT cheetah, an electrically-powered quadrupedal robot for high-speed running. J. Robot. Soc. Jpn. 32, 323–328. 10.7210/jrsj.32.323

[B28] Reiley C. E. Akinbiyi T. Burschka D. Chang D. C. Okamura A. M. Yuh D. D. (2008). Effects of visual force feedback on robot-assisted surgical task performance. J. Thorac. Cardiovasc. Surg. 135, 196–202. 10.1016/j.jtcvs.2007.08.043 18179942 PMC2674617

[B29] SoftBank Robotics (2017). Robot Evolution. Press Conf. New Approaches SoftBank Robot. Available online at: https://www.softbankrobotics.com/jp/set/data/news/info/20171120a/20171120_01.pdf (accessed August 24, 2020).

[B30] Tachi S. Tanie K. Komoriya K. Kaneko M. (1985). “Tele-existence (I): design and evaluation of a visual display with sensation of presence,” in Theory and Practice of Robots and Manipulators, eds. A. Morecki, G. Bianchi, and K. Kȩdzior (Boston, MA: Springer), 245–254. 10.1007/978-1-4615-9882-4_27

[B31] Talasaz A. Trejos A. L. Patel R. V. (2017). The role of direct and visual force feedback in suturing using a 7-DOF dual-arm teleoperated system. IEEE Trans. Haptics. 10, 276–287. 10.1109/TOH.2016.2616874 28113408

[B32] The Japan Institute for Labour Policy Training (2016). Labor Situation in Japan and its Analysis: General Overview 2015/2016. Tokyo: The Japan Institute for Labour Policy and Training. Available online at: https://www.jil.go.jp/english/lsj/general/2015-2016.html

[B33] Tobergte A. Helmer P. Hagn U. Rouiller P. Thielmann S. Grange S. (2011). “The sigma.7 haptic interface for MiroSurge: A new bi-manual surgical console,” in IEEE International Conference on Intelligent Robots and Systems (San Francisco, CA), 3023–3030. 10.1109/IROS.2011.6048043

[B34] Wijntjes M. W. A. Sato A. Hayward V. Kappers A. M. L. (2009). Local surface orientation dominates haptic curvature discrimination. IEEE Trans. Haptics. 2, 94–102. 10.1109/TOH.2009.1 27788100

[B35] Williams L. E. P. Loftin R. B. Aldridge H. A. Bluethmann W. J. (2002). “Kinesthetic and visual force display for telerobotics,” in Proceedings 2002 IEEE International Conference on Robotics and Automation (Washington, DC), 1249–1254. 10.1109/ROBOT.2002.1014714

[B36] Yamamoto A. Cros B. Hashimoto H. Higuchi T. (2004). “Control of thermal tactile display based on prediction of contact temperature,” in Proceedings - IEEE International Conference on Robotics and Automation (New Orleans, LA), 1536–1541. 10.1109/robot.2004.1308042

